# Advanced maternal age and risk perception: A qualitative study

**DOI:** 10.1186/1471-2393-12-100

**Published:** 2012-09-19

**Authors:** Hamideh Bayrampour, Maureen Heaman, Karen A Duncan, Suzanne Tough

**Affiliations:** 1 Department of Pediatrics, Faculty of Medicine, University of Calgary, Alberta Centre for Child, Family & Community Research- Child Development Centre, c/o 2888 Shaganappi Trail NW, Calgary, AB, T3B 6A8, Canada; 2 Faculty of Nursing, CIHR Chair in Gender and Health, University of Manitoba, Room 268 Helen Glass Centre for Nursing, 89 Curry Place, Winnipeg, MB, R3T 2N2, Canada; 3 Family Social Sciences, Faculty of Human Ecology, University of Manitoba, 314B Human Ecology Building Winnipeg, Manitoba, MB, R3T 2N2, Canada; 4 Alberta Innovates Health Solutions Health Scholar and Professor in Department of Pediatrics and Community Health Sciences, University of Calgary, Alberta Centre for Child, Family & Community Research- Child Development Centre, c/o 2888 Shaganappi Trail NW, Calgary, AB, T3B 6A8, Canada

**Keywords:** Advanced maternal age, Risk perception, Qualitative study

## Abstract

**Background:**

Advanced maternal age (AMA) is associated with several adverse pregnancy outcomes, hence these pregnancies are considered to be “high risk.” A review of the empirical literature suggests that it is not clear how women of AMA evaluate their pregnancy risk. This study aimed to address this gap by exploring the risk perception of pregnant women of AMA.

**Methods:**

A qualitative descriptive study was undertaken to obtain a rich and detailed source of explanatory data regarding perceived pregnancy risk of 15 women of AMA. The sample was recruited from a variety of settings in Winnipeg, Canada. In-depth interviews were conducted with nulliparous women aged 35 years or older, in their third trimester, and with singleton pregnancies. Interviews were recorded and transcribed verbatim, and content analysis was used to identify themes and categories.

**Results:**

Four main themes emerged: definition of pregnancy risk, factors influencing risk perception, risk alleviation strategies, and risk communication with health professionals.

**Conclusions:**

Several factors may influence women's perception of pregnancy risk including medical risk, psychological elements, characteristics of the risk, stage of pregnancy, and health care provider’s opinion. Understanding these influential factors may help health professionals who care for pregnant women of AMA to gain insight into their perspectives on pregnancy risk and improve the effectiveness of risk communication strategies with this group.

## Background

In the past three decades, an increasing proportion of women have delayed childbearing for educational, social, and economic reasons [1,2]. Pregnancy at advanced maternal age (AMA), defined as age 35 years or older, is associated with several adverse pregnancy outcomes including preterm birth, low birth weight, still birth, chromosomal defects, labor complications, and cesarean section [3-7]; therefore, it is considered to be a “high risk” pregnancy. However, little is known about how women of AMA perceive their pregnancy risk. Understanding perception of pregnancy risk is important, because it can affect women’s health care use, motivations to seek care, pregnancy and labor decisions, adherence to medical recommendations, and health behavior [8-10]. Risk perception is also incorporated as a key concept in constructing several theories of health behavior such as the Health Belief Model [11], Protection Motivation Theory [12], and Prospect Theory [13].

The concept of risk at AMA may be composed of two components: the physiological challenges because of an aging reproductive system, and the social discourse of risk and timing of childbearing [14]. Most previous work on risk perception at AMA has focused on increased risk of genetic abnormalities, and the general concept of pregnancy risk perception has received less consideration. Current knowledge of risk perception at AMA is based on a few studies that most of them primarily focused on pregnancy experiences, in which risk perception was discussed as part of the pregnancy and birth experience. For instance, Windridge and Berryman (1999), in a study of 107 British women’s experiences of giving birth at AMA, explored risk perception and reported that women of AMA may perceive a higher risk for their babies during labor because of their older age [15]. Another notable study on this topic is an Australian qualitative study of 22 prim gravid women of AMA that was initially conducted to understand the experiences of women of AMA [16]. Later, in 2007, Carolan and Nelson conducted a secondary analysis of the qualitative data and identified four themes central to perception of pregnancy risk including "realizing I was at risk," "hoping for reassurance," "dealing with uncertainty," and "getting through it/negotiating risk"[14], p.540. They reported high levels of concern and anxiety among participants, in spite of these women having healthy pregnancies, and concluded that the notion of risk may have negative impacts on these women. A qualitative study by Saxell (2003) primarily focused on studying the understandings and beliefs about the risks associated with the pregnancies of ten women of AMA. Saxell reported that these women found being labeled as high risk very challenging; hence, several participants rejected this labeling as part of their coping mechanism. This study had a number of limitations such as retrospective data collection (36 months after delivery) and its unique population (participants who chose midwifery care), which should be considered in the interpretation of the results.

The focus of previous studies has been on describing the impact of being labeled as high risk on pregnant women, rather than exploring and elaborating on the process of risk appraisal by these women. Understanding women’s perception of pregnancy risk is essential in developing more effective risk communication approaches. Considering the lack of literature in this area and limitations associated with previous studies, we conducted a mixed methods research study to further existing knowledge on risk perception and its contributing factors. Mixed methods research is "research in which the investigator collects and analyzes data, integrates the findings, and draws inferences using both qualitative and quantitative approaches or methods in a single study or a program of inquiry" [17], p.4. Mixed methods research gives the researcher an ability to use both numbers and words and combine inductive and deductive thinking to address the research problem [18]. Our study was composed of both qualitative and quantitative components and using a mixed methods approach, we compared or integrated the factors contributing to perception of pregnancy risk (determined by multiple regression analyses) with the women's perspectives (obtained from 15 qualitative interviews) to enhance current knowledge on this topic. The sample for the mixed methods study consisted of 159 participants (105 women aged 20-29 years and 54 women aged 35 years or older). In the quantitative component, we compared perception of pregnancy risk in nulliparous women aged 35 years or older (n = 54) with that of nulliparous women aged 20-29 years (n = 105) [19]. In the quantitative component, we also developed a conceptual framework to explore factors contributing to risk perception among nulliparous women (N = 159) [20]. The study reported here is the qualitative component of this mixed methods study. The objectives of this qualitative study were to explore how nulliparous women of AMA evaluate and define their pregnancy risk and to arrive at a detailed understanding of their risk perception. The following research questions were explored: 1. How do nulliparous women of AMA define pregnancy risk? 2. Why do women of AMA feel that their pregnancy is, or is not, at risk? Which factors influence perception of pregnancy risk? 3. What impact, if any, did being labeled high risk have on these women’s lives and behaviors? 4. What are the experiences of women of AMA with risk communication from health care providers during pregnancy? How do women feel their perceptions of risk differ from those of their health care providers, if at all?

## Methods

A qualitative descriptive study was undertaken to obtain a rich and detailed source of explanatory data regarding risk perception from women of AMA. As described by Sandelowski (2000), a qualitative descriptive study provides "a comprehensive summary of an event in the everyday terms of those events" [21], p.336. The qualitative descriptive method is a distinctive and valuable technique and offers a direct description of phenomena, which is still interpretive [22]. As Sandelowski (2000) stated, this kind of research can address both descriptive and interpretive validity to account for participants' observations and their understandings of these events.

Participants were chosen purposefully from among the participants in the quantitative component of the mixed methods study. Criteria for participation in the larger mixed methods study were women with a singleton pregnancy, ability to speak, read, and write English, and gestational age of 28 weeks or greater. Any known severe psychological disorder was an exclusion criterion. Data were collected over 14 months, from (December 2009 to January 2011) from selected physicians' offices and outpatient prenatal care clinics at two tertiary hospitals in Winnipeg, Manitoba.

In purposeful sampling, participants are intentionally selected according to the needs of the study [23]. The researcher asked selected women aged 35 years or older who had participated in the quantitative component of the study if they were willing to participate in the qualitative component. Participants were selected to ensure diversity in demographic and obstetric characteristics, most notably gestational age and pregnancy complications. In addition, participants were recruited to reflect a range of scores using the Perception of Pregnancy Risk Questionnaire [24] used in the quantitative component, to ensure inclusion of participants with different levels of perception of pregnancy risk.

Potential participants were provided with a written and verbal explanation of the study. Each participant signed a consent form. A mutually convenient time and place were arranged to conduct the interviews. In-depth, semi-structured, face to face interviews, using an interview guide, were conducted by the researcher from January 2010 to January 2011. The interview guide was developed by the investigators and key areas explored included decision making about timing of childbearing, women’s understandings about pregnancy after age 35, perceived pregnancy risk and its impact on woman’s feelings and thoughts, partner’s perspectives about pregnancy, and women’s experiences with the health care system. All interviews were audio-recorded and transcribed verbatim. In qualitative inquiry, sample size relies on the concept of “saturation” which is the point at which no new information or themes are observed in the data [25]. Saturation was reached after interviewing 15 participants.

Data analysis was carried out concurrently with data collection. Transcripts were analyzed using a content analysis technique [26]. Content analysis is a systematic and replicable method used for condensing many words of text into fewer content categories based on explicit rules of coding [27]. As mentioned earlier, this study was part of a mixed methods research study in which several instruments were administered to women participating in the quantitative component. In this report, selected quantitative measures including the Perception of Pregnancy Risk Questionnaire (PPRQ) [24], perceived internal control [28], and pregnancy-related anxiety [29] scores have been reported to enrich information about participants and enhance the interpretations of the findings. The scores on these scales were divided into tertiles representing low, middle, and high scores, based on the responses of the women aged 35 years or older in the larger mixed methods study (n = 54) (Table 1). Table 2 illustrates the number of participants in each tertile from the qualitative component.

**Table 1:** Classifications of the PPRQ, Pregnancy-related Anxiety, and Perceived Control (internal) Scores in Tertiles Using Data for Women of AMA from the Quantitative Component of the Advanced Maternal Age and Risk Perception Study (n = 54)

**Instrument**	**Minimum and Maximum Scores**	**Low Tertile**	**Middle Tertile**	**High Tertile**
Perception of Pregnancy Risk Questionnaire	3.56-69.44	<18.68	18.68-35.33	>35.33
Pregnancy-related Anxiety	1.10-3.20	<1.71	1.71-2.00	>2.00
Perceived Internal Control	16.00-36.00	<24.01	24.01-28.00	>28.00

**Table 2:** Frequency of Participants in each Tertile in the Qualitative Component (N = 15)

**Instrument**	**Low Tertile**	**Moderate Tertile**	**High Tertile**
Perception of Pregnancy Risk Questionnaire	6	5	4
Pregnancy-related Anxiety	5	6	4
Perceived Internal Control	4	8	3

According to Davies and Dodd (2002), rigor refers to the reliability and validity of qualitative research; however, it does not necessitate the sense of replicability of the research in different situations that is common in quantitative research [30]. In the current study, a validation process was performed during and after conducting the interviews using two strategies. In the first method, member checking was used in which during each interview, the researcher (HB) verified the accuracy of her interpretations with participants by asking further questions. After completion of data analysis, interpretations of data were shared by twelve participants who had previously agreed to review and verify findings of the study. Second, the results were confirmed with peer review and debriefing through regular meetings with one of the co-investigators (MH) to achieve a jointly developed interpretation of the data. Dependability was obtained through use of an audit trail. In this strategy, contextual information and participant’s reflections were documented. The ways to address confirmability in this study were using direct quotes and confirming the findings with participants during interviews.

This project was reviewed and approved by four committees prior to commencing data collection: the University of Manitoba Education/Nursing Research Ethics Board, the Health Sciences Centre Research Impact Committee, the St. Boniface Hospital Research Review Committee, and the Winnipeg Regional Health Authority Research Review Committee.

## Results

The sample consisted of 15 nulliparous women. The age range of participants was 35 to 44 years with a mean of 37.6 years. The majority of women were highly educated with a mean educational level of 18.7 years. All participants were married or living common-law. All except one had been working during pregnancy. Over half (53.3%) of the women reported an annual family income of $100,000 or over. All participants were in the last trimester of their pregnancies, with a mean gestational age of 35.6 weeks (ranging from 32 to 40 weeks). All participants except one considered their pregnancy to be planned. Five participants had one or more previous miscarriages and three participants reported use of fertility medications to become pregnant. Participants delayed their childbearing for a variety of reasons. The primary reason for some women was lifestyle choices (n = 6). This group of women described being focused on their career, education, or travel, or indicated that they did not feel that they were ready to have children until they reached an older age. Delayed marriage was the primary reason for six other participants. Finally, three women identified infertility issues as their primary reason for postponed childbearing. It should be noted that infertility was also reported among participants in the delayed marriage and lifestyle choices groups, however it was not their prime reason for delayed childbearing.

The findings of this study were categorized into the following themes: definition of pregnancy risk, factors influencing risk perception, risk alleviation strategies, and risk communication with health professionals.

### Definition of pregnancy risk

The first theme describes participants’ understandings of pregnancy risk and explains how they defined risk. Most participants acknowledged that pregnancy risk increases as maternal age rises; however, a number of women felt that the risk had been overestimated. For example, one of the participants stated that "those numbers don’t matter," (Participant 1) and another mentioned that "it’s an overreaction, frankly" (Participant 11). Women’s understandings of risks associated with AMA were reflected most often in comments about their awareness of increased risk of infertility issues and genetic abnormalities, particularly Down syndrome. Fertility problems associated with delayed childbearing were an immense concern for most women rather than pregnancy-related issues. In this regard, most of the women who delayed marriage reported that they decided to become pregnant soon after marriage to minimize the effect of age factor on their fecundity.

It was noted that women considered pregnancy as more of a societal issue rather than merely a biomedical matter, in which personal, interpersonal, and societal elements were significant. For our participants, the meaning of risk was broader than medical issues and included the extent of their support network, their ability to control situations, and whether they had a secure relationship, a planned pregnancy, a flexible job or healthy lifestyle and behavior. These considerations were also evident in decisions regarding timing of pregnancy by participants. The following quote is an example of the broadness of risk definition by one participant with an uncomplicated pregnancy:

""A high risk pregnancy to me would have been if I had no control over my work environment . . . So even just the ability to be able to work from home . . . if I was working in more a violent situation, if I was in a bad relationship I think that would make me more of a high-risk pregnancy, than my personal age or my personal health." (Participant 13)"

In the following quote, a 39 year-old participant stated on the importance of having a stable relationship and a healthy lifestyle:

""I think you; you have to factor in your physical condition . . . [that] you’re in good health and you’re taking care of yourself and you’re planning on getting married and you go and get your physicals . . . I see a lot of younger girls that are pregnant and they’re drinking bottles of coke and they’re drinking coffee and they’re drinking, you know they’re eating McDonald’s and I’m like that hasn’t been my pregnancy, you know I’ve cut out sugar and I’ve never drank coffee so I don’t worry about that. " (Participant 1)"

A 36 year-old participant with gestational diabetes and a moderate perceived risk score commented:

""I had this, this calm, calm feeling inside of me that everything was going to be okay . . . having a husband that is loving, caring, supportive and you know [is] my best friend, so that really really helps a lot, and having friends, you know friends to support to, to help you, to talk with you if you have any need and we have so many people caring about us, so um . . . having a whole community to, to be there for you if you need." (Participant 12)"

### Factors influencing risk perception

The magnitude of the perception of pregnancy risk differed among our participants. During data analysis, nine factors were identified that influenced women's perception of pregnancy risk including: cognitive heuristics of availability and similarity of the risk, predictability of the risk, health status, pregnancy complications, gestational age, perceived control, poor fertility history, anxiety, and the health care provider’s opinion.

#### Cognitive heuristics

Women’s understandings of risk were mostly based on their experiences of real cases, and they relied on these data in assessment of their own pregnancy risk:

""From my experience in Europe, where most women are having their children in their thirties and not their twenties and, to be honest, I didn’t, and I lived there for a long time, I didn’t see the amount of Down’s syndrome or other physical abnormalities that you see here." (Participant 11)"

Most participants had an example of pregnancy at AMA among one of their family members, friends or colleagues. Availability of a positive example or a favorable family history of fertility at AMA was often reported as a reassuring factor that increased women's perceived ability to conceive at AMA and to have a successful pregnancy. These participants often perceived their pregnancy as being low risk.

""I know a lot of women that are having their first child in their forties . . . and everything has worked out fantastic, so I always looked at them as sort of my mentors or role models." (Participant 5)"

""Both like my mother had me when she was 36, I think, and my partner’s mother had her last one when she was 41, so it’s, it doesn’t seem that abnormal in my [family] . . . And I think maybe that . . . made me feel less risky as well cause I had, you know, generations before me that had late babies and with no complications." (Participant 8)"

On the other hand, women who were familiar with the risks associated with older maternal age, through personal or vicarious experiences, expressed more concerns and perceived a higher risk for their pregnancies. One of the participants with a fairly healthy pregnancy and a high perception of pregnancy risk score commented:

""A friend of mine got pregnant; I think she was 36, and she did have a baby who had so many problems that they were surprised the baby’s heart was even still beating in her womb, so she ended up terminating that pregnancy, and she’s still broken up about . . . so I was debating this . . . So yeah I did have that one friend, and of course, the woman who told me about her near death experience in labor." (Participant 4)"

"Availability of risk" is a cognitive heuristic that refers to "a cognitive shortcut used for judging the probability of an event by the ease with which examples of the event come to mind" [29], p.248. Based on participants’ explanations, this concept appeared to be very important in risk appraisal. One participant with a high perception of pregnancy risk score explained: "if it [risk] happens to other people, then it could happen to me."

"Similarity" is another cognitive heuristic and reflects "a cognitive shortcut used for judging the probability of an event by its similarity to events with comparable features" [29], p.248. Women often assessed their pregnancy risk by comparing their own characteristics with those who developed a pregnancy complication. The following comment from a 37 year-old participant is an example of using the similarity heuristic in risk appraisal:

""I had that friend who was 36 and had a baby who had Trisomy 18, and she felt that it was because of her age . . . but she is not as healthy as I am. She actually may have an alcohol problem, and she’s taken a lot of medications in her life for depression and all kinds of things, and she doesn’t really work out very much, and she just wasn’t as healthy [as I am]."(Participant 4)"

#### Predictability of the risk

An expected and predictable pregnancy complication was perceived as a lower risk situation than a non-predicted and unexpected problem. One participant with a low perceived risk score, who had severe morning sickness in early pregnancy, described it as a “typical pregnancy symptom” that could happen in “every pregnancy.” On the other hand, another participant with a high perception of risk score and a low perceived control score, who had to stop working for an unexpected, threatened premature labor stated:

"“It’s been challenging, so it’s not what I expected . . . I did not expect to feel an almost arthritic pain the last two months, I didn’t expect that morning sickness would be 24 hours a day for five months instead of three, neither of us were expecting this . . . I was expecting to work until my due date and come to the hospital and you know I was ready to be like every other mom, well most other moms where you work till the end and sometimes you just go from work straight to the hospital in labor.” (Participant 6)"

A 37 year-old participant with a high perception of risk score who was diagnosed with gestational diabetes explained that:

""Both of my parents have adult onset diabetes, and my mother had gestational diabetes with my brother when she was roughly the same age as me, and so I, pretty much expected to have gestational diabetes, so I was careful right from the beginning, making sure that I was exercising and trying to eat healthfully and so on . . . so I was cautious about that . . . [however] I was surprised to find that I had needed the insulin for it as opposed [to] control by diet; that was a bit of a shock. It was a shock to discover that it was that severe to begin with and that I would need the insulin." (Participant 9)"

#### Health status

Most women in our study considered themselves healthy and felt that they had control over their physical health. Good health was frequently identified as a factor that would decrease risk when participants assessed their pregnancy risk in relation to age. They often emphasized their good health as a balancing capacity in pregnancy risk estimation. A 35 year-old participant at 30 weeks of gestation with a low perception of risk score commented:

""Because I was, I’m always, I’ve always been a quite a healthy person, it, it never really concerned me that much that I was older and having a baby." (Participant 8)"

#### Pregnancy complications

Among our participants, three women had gestational diabetes, one had vaginal bleeding before 20 weeks, and one had both vaginal bleeding before 20 weeks and threatened preterm labor. Based on our findings, pregnancy complications had some influence on the perception of pregnancy risk; depending on the type of complication, its impact on the woman's life, and the degree to which the woman felt that complication had been controlled. For instance, the woman with threatened preterm labor who had to quit work expressed a high risk perception. On the other hand, the other three women, who described their gestational diabetes as a well-controlled situation, had low to moderate perception of risk scores. The following statement was made by one of the participants who had a moderate perceived risk score:

""I’ve developed gestational diabetes in the end but that's probably due to the fact that I already had polycystic ovary syndrome, so there was already insulin resistance . . . there was nausea initially but no vomiting, um a lot of weight gain, but again that's the insulin resistance, so it’s been an easy pregnancy, I suppose." (Participant 11)"

Women with pregnancy complications often had higher pregnancy-related anxiety scores. One of the participants with gestational diabetes expressed concerns regarding having a larger baby than average and also long-term consequences of diabetes:

""The first week [after diagnosis] I was very very sad, I was very sad because I, I was concerned, you know, having a big baby and, and he has any complication [in] the pregnancy, um I was very sad for him that I may be passing this gene." (Participant 12)"

#### Gestational age

Most participants reported that they felt more relaxed about risk and developing pregnancy-related problems as their pregnancy progressed. The participants described pregnancy as having several milestones, and completing each phase seemed to increase their confidence in having a healthy pregnancy:

""As time went on, it got a little bit easier to feel like I wasn’t at as much risk in terms of the pregnancy." (Participant 7)"

""As I get closer [to delivery] and I haven’t had any problems I’m getting more and more confident and I feel a little bit better." (Participant 1)"

Toward the end of pregnancy, the meaning of older age as a risk factor for most women with uncomplicated pregnancies remained simply a number. Some women used phrases such as "it was just that number and that's it!"

#### Perceived control

The majority of women in our study perceived themselves to be prepared to accept child raising responsibilities. Participants’ comments reflected that most of them had a good sense of internal control, both over their life situations and their health. Considering the pregnancy-related anxiety and risk perception scores of participants and their explanations, it was evident that women with high perceived control had lower perceived risk. The following quote was expressed by a 39 year-old participant who had a high perceived control score and a low perception of risk score:

""I just [know] the way my body responds to things . . . I understand my body, I’m very in tune with how my body works and I can always tell when something’s not right." (Participant 1)"

Conversely, low perceived control was often observed in conjunction with high perceived risk. Sometimes the woman’s religious beliefs changed the equation. The following statement was made by a 44 year-old participant who had both low perceived control and low perceived risk scores:

""Both of us felt strongly that . . . um you know we believe strongly in God and that he was in control." (Participant 7)"

#### Poor fertility history

A number of participants mentioned that they lost a sense of control over their bodies and their own health to a great extent after enduring infertility experiences. For some women a history of infertility or miscarriage led to increased risk perception in pregnancy. A 37 year-old woman with a high risk perception score and a history of infertility commented:

"“I planned to get pregnant . . . and I wasn’t able to, like the first thing that came to my mind, I’m not a life giver, it’s not because you wanted to get pregnant at this month you’ll get pregnant, I am not in control. If I’m not given that then I can’t do, it’s beyond my powers.” (Participant 15)"

The emotional impact of previous fertility issues on the current pregnancy was expressed by words such as having worry, stress, and fear. For example, a 37 year-old woman with a previous miscarriage who had a high perception of pregnancy risk score stated:

"“I wasn’t at [risk] first, but then after I lost the first baby, I thought well maybe this is because I’m old . . . I was stressed, I didn’t really deal with it.” (Participant 4)"

A 44 year-old participant believed that infertility treatments had a negative impact on her physical health. This belief was also tracked in other participants' explanations with infertility experiences:

"“I think what plays into how I perceive my pregnancy is, is not just my age, it’s the journey I took to get here, it was because it took four years to get here . . . and it was a hard four years. My body wasn’t [at risk], I wasn’t, when we first started trying I was extremely healthy and in shape and, and felt ready, and then as I went through all the fertility procedures my health, it affected my health . . . and I didn’t feel myself, I didn’t feel as strong as I was, so coming into a pregnancy now where I was coming off of all that fertility stuff, it made me feel like I might be at a higher risk." (Participant 7)"

#### Anxiety

Although most participants in this study expressed some degree of concern for the health and well-being of their babies, only a few women were very anxious. Nevertheless, we found that these women often did not use "anxiety" as a term to describe their anxious feelings. For instance, the following quote comes from a 39 year-old participant who had a high anxiety score:

""There still is a little bit in the back of my mind, once the baby is born . . . is the baby going to, is everything going to be okay with the baby and through delivery and through labor? So there is a little bit, not anxiety, I wouldn’t say anxiety, but a little bit of concern." (Participant 5)"

"Being emotional" was another term that a 35 year-old participant with a high anxiety score and moderate perception of risk score used to express her feelings:

""I’m a little bit emotional, I think that's my biggest, been my biggest struggle throughout the entire pregnancy is my emotional state. First with being so worried and then hormones, I wasn’t, I was depressed, and now I’m having trouble sleeping, which is causing a lot of frustration, and I’m really uncomfortable." (Participant 2)"

According to participants' explanations, waiting for screening results, bed rest and limiting physical activities due to pregnancy complications (e.g., morning sickness) contributed to their risk perception. One participant with a high perceived risk score and a high anxiety score who experienced morning sickness in early pregnancy explained:

""I didn’t feel very good for many months, so I couldn’t eat, I was barfing a lot, so that probably added to my stress being home all day cause I couldn’t work and not being able to eat, so that always, you know, increases your stress, and it lowers your resistance too." (Participant 4)"

Feeling that this pregnancy might be their last chance to have a biological child might increase anxiety for these women and add to the dilemma. A 40 year-old participant with a complicated pregnancy who had a high perception of risk score and a low perceived control score explained:

""High risk mostly is, or my perception of high risk is if something happened to myself or my child, this may be my only chance to be pregnant and deliver a child." (Participant 6)"

#### Health care providers’ opinion

Health care providers’ perspectives about risk and their reaction to, and interpretation of, risk had a considerable influence on the women's understandings and assessments of their own risk. Participants' explanations revealed that health care providers’ opinions, as experts in risk assessment, were viewed as very valuable and relied upon by women. Although care providers do not usually disclose their personal opinion, women may intuit it by observing care providers’ reactions and body language. Women may include this information in determining of their risk levels, especially if there is an uncertainty associated with the level of vulnerability. As a 37 year-old participant with a low perception of risk score and a low anxiety score remarked:

""My family doctor called me to tell me that the baby has to have an ultrasound after a month, but the doctor here doesn’t seem, doesn’t seem bothered by it, so I don’t think it’s got anything to do with the baby having, like, mental or physical disability of any sorts; I think it’s just more, just something for them to look at." (Participant 13)"

Taking into consideration that several participants in this study reported that they did not have major discussions with their health care providers about risks associated with AMA, it might give these women a degree of confidence that there are no serious concerns.

### Risk alleviation strategies

Several strategies were reported by the participants to help them alleviate and/or cope with their pregnancy risk. This process, for our participants, involved educating themselves and engaging in a healthy lifestyle, undertaking reassuring surveillance tests, overlooking the risk, relying on religious beliefs and hope, and balancing their risk by emphasis on the positive social aspects of AMA.

#### Educating themselves and engaging in a healthy lifestyle

Some participants spoke of gathering information to know what actual risks are and to be prepared. Participants with low perceived risk scores and high or moderate perceived control scores often reported that they had prepared themselves for pregnancy complications to "not be surprised by risk" (Participant 1). This 39 year-old participant with a high perceived control score who perceived a low risk for her pregnancy stated:

""I’m healthy, I’m fine and I’ve prepared myself, I’ve educated myself . . . Yeah I think it would be less stressful [if I were younger] because I wouldn’t have to educate myself so much. I would have educated myself but I feel like I had to do that extra little bit of research because I was in that high risk group, I was in that you know “more chance of something going wrong group.” So I educated myself a little bit more." (Participant 1)"

These participants were also focused on improving their lifestyle and engaging in healthy behaviors to protect their pregnancies.

#### Reassuring surveillance tests

Because most women were aware of the association between genetic problems and older maternal age, receiving reassuring screening test results was reported as a relieving factor. A desire to seek actual and tangible signs of the baby's health to confirm that "it's a normal baby" was evident in the interviews:

""When I had one [ultrasound], and I saw that everything was okay, he was developing okay, then I had the tests done, the blood test, I said okay, he’s a normal baby, so I stopped worrying." (Participant 12)"

Some participants chose to take screening tests as part of their preparation to deal with anticipatory issues and to decrease concerns related to fetal health. Based on several participants explanations, undertaking screening tests was a reflection of their desire to plan and be prepared:

""We just wanted to do it for our own sake of mind, just for our own peace of mind. I like planning my life, I like preparing for things, I like being educated and ready for whatever, I wanted to know, if I’m going to have a baby with Down’s syndrome. I want to know so that I’m prepared, so that I can educate myself as to what this baby’s needs are going to be. At least if you’ve prepared a little bit, you might be able to handle it differently." (Participant 1)"

#### Overlooking (ignoring) the risk

Some participants reported an inclination to disregard the risk to avoid excessive stress and anxiety. In fact, overlooking the risk was a very common approach among our participants, even among those who educated themselves and were engaged in a healthy lifestyle:

""I’ve been trying not to let research or other reports or what I read influence my way of thinking . . . I know they exist, I’m not saying that ignorance is bliss, but I just don’t focus on it." (Participant 1)"

The following quote from a 44 year-old participant who had both a low perceived risk score and a low perceived control score describes this approach. She and her partner decided not to undergo genetic screening tests.

""I did a fair bit of reading but then at the same time I also got to a point where when I was pregnant I put the reading aside because . . . I just found it was starting to increase a lot of anxiety about all the things that could go wrong or be wrong . . . considering my age and considering the risks . . . I didn’t want to go into the constant anxiety about it . . . we didn’t want to be ignorant about it, like we, we did all our research and awareness, uh like I said, we made sure we were eating healthy, but I mean other than that, there’s [not much] else you can do, you can’t, it’s out of our control . . . once we talked through some of those anxieties, we took all the necessary precautions to make sure that it was a healthy pregnancy and other than that, the rest is out of our hands." (Participant 7)"

#### Religious beliefs and hope

Religious beliefs and hope were other alleviating factors described by some participants. They reported that these beliefs helped them to stay calm and feel that they were not alone. A woman with a high perceived risk score and a low anxiety score commented that:

""I think my, my faith in, in God somehow kind of like helps me go to sleep at night, you know like I mean I pray…" (Participant 15)"

Another participant with a high perceived risk score and a moderate anxiety score expressed that her religious beliefs were important in how she dealt with the risk. This participant had a low perceived control score and decided not to undergo genetic screening tests.

""I think that's a matter of our faith and our belief, um that you know, I mean, if God created this child, if God chooses to take this child, then that's his decision to make and not ours, so we’re not playing God by taking that child’s life." (Participant 9)"

#### Emphasis on positive social aspects of AMA

A tendency to emphasize positive aspects of pregnancy at older age emerged during the interviews. From some of our participants' perspectives, an older age improved their readiness to be a parent by having an established relationship and career, being mature both emotionally and personally, and developing problem solving skills through having various life experiences. Our participants weighed these advantages against biomedical risk in their risk assessment. The following quotes are from participants who had moderate perceived risk scores:

""There’s a risk, and it’s high risk. But if, if you ask me if I would prefer being pregnant [at this age] or at 24, I would say right now. I was not emotionally prepared at that time to have a baby . . . I was not strong, a strong woman that I’m right now, I have much more to offer my son [now] than if I was young." (Participant 12)"

""I do think that I would probably have more energy ten years ago, but at the same time, emotionally, I wasn’t ready. I was immature and irresponsible still ten years ago. Now I’m more mature and more emotionally ready for it." (Participant 2)"

### Communication with health professionals

Communication about risk from health professionals mostly focused on recommending screening tests. Participants' explanations reflected that, in the majority of cases, there was not a discussion about other age-related risks. On the other hand, most women preferred not to initiate risk communication. They reported receiving extra information about risk associated with older age as pointless and most likely stressful and anxiety provoking, because they knew they would not be able to change their risk factor; age:

""I was aware, and I knew, and he was extremely open to questions; he’s very approachable, so I knew that if I had concerns, or if I had questions about it, I could talk to him, but I didn’t feel the need." (Participant 7)"

Some women reported having negative communication with health professionals, as reflected by the following quote from a 35 year-old participant with a low risk pregnancy:

"“Um the lady that gave me the ultrasound I think she was judging me a bit for being older, cause she did ask me you know if I had taken all the extra screening and I told her no and I don’t think she was too impressed by that . . . and then . . . she’s like I think people that have babies when they’re really old that's just unethical . . . and so I definitely got from her that uh you know she was judging me a bit for being older . . .” (Participant 8)"

Some participants found risk discussions with their health care provider stressful. In reviewing participants’ statements and their anxiety scores, it was apparent that anxiety may influence the way a pregnant woman interpreted risk information and the contents of risk communication. The following quote was offered by a 37 year-old participant who had both a high anxiety score and a high perceived risk score:

""I was scared after talking to the genetic counselor for sure. The nurse with my GP always mentioned it, every visit, she would say, ‘well you do have high risks because of your age.’ Every week. I know my age; you don’t have to keep driving the point home . . . Cause I had a lot of fear actually, when I was meeting with the genetic counselor and with the nurse at my family doctor’s office. I was strongly encouraged to go for genetic counseling because of my advanced age. The terminology that the doctors and nurses and everybody [used], advanced age, advanced age, I’m not eighty, but okay, I suppose I’m over 35 . . . And they would show me charts, like, oh this is how your risk increases once you’ve reached 35, and you kind of feel a fear that you know something’s going to go wrong cause you’re over 35. And the genetic counselor drew all these charts and showed me my risk. But after the genetic counseling and the results came back from all the tests, it turns out I have an extremely low risk, so I’m not sure why they try to scare you like that." (Participant 4)"

## Discussion

The definition of risk for women of AMA, similar to that of pregnant women of other ages [31], had a broader scope than merely medical risks or physical challenges and was influenced by various social and personal characteristics. The findings described above elucidated that this definition also incorporates consideration of the extent of the woman’s support network, her ability to control situations, and whether she had an established relationship, a planned pregnancy, a flexible job or healthy lifestyle and behavior. These findings support the position that approaches to understanding perception of health risks should be comprehensive and broad [8] and suggest that risk communication will benefit from including women's criteria for defining risk.

As is the case in previous research [32], pregnancy at AMA was frequently acknowledged by women to be high risk due to an increased risk of genetic abnormalities; however, other medical risks associated with AMA received less consideration. In our study, the "information quests"[32], in which women sought out a wide range of information, were commonly reported in the preconception period. After becoming pregnant, however, women mostly limited seeking information to avoid increased anxiety and were focused on risk reducing behavior instead. In contrast to findings of Carolan & Nelson (2007) who reported that their Australian participants realized they were at higher risk only after becoming pregnant and through communication with their health care providers, women in our study described risks associated with AMA as "common knowledge in society." This finding is consistent with the finding of another Canadian study [33].

Findings elucidated that nulliparous women aged 35 years or older were not a homogenous group in their pregnancy risk assessment. The data described above suggest that the perception of pregnancy risk may be a result of interactions among several factors including physiological and psychological elements, characteristics of the experienced risk, and feedback from health care providers. Despite the fact that our respondents were mostly aware of risks associated with pregnancy at age 35 years or older, the majority of them did not consider themselves to be high risk. This apparent disconnect can be explained by the fact that people typically tend to rate their personal risk lower in comparison to general risk [34]. This underestimation of personal risk or "unrealistic optimism" [35], might be related to one's perceived control over a potential risk [34]. A link has been identified between perceived control and risk perception in previous research [36]. In our study, those participants who believed they had good control over their physical health also perceived a lower risk for their pregnancy. It was also noted that a woman's perceived control over her health may be important in engaging in various behaviors to maintain the balance between risk and health. To support this point, a relationship between perceived control and health service utilization has been documented in previous research [37].

Previous prenatal loss was recently documented as a predictor of depression and anxiety in subsequent pregnancies, independent of other psychosocial and obstetric factors [38]. Our findings highlighted that having a poor reproductive history contributes to increased perceived risk of pregnancy. Failure to become pregnant in initial attempts or previous loss of pregnancy may have threatened these women's beliefs in their abilities to manage their own health and may lead to increased anxiety and higher risk perception. This statement is supported by Campbell, Dunkel-Schetter, & Peplau (1991) who reported that infertility may be perceived as a threat to an infertile person's life's goals, contributing to a low perception of control [39]. Based on our data, pregnancy complications could alter risk perception depending on the type of complication, its manageability, and its consequences for a woman's daily life. Alternatively, good physical health and engaging in healthy behaviors and lifestyles were perceived as risk alleviating factors. This finding echoes that of Gerend et al. (2004) who reported that personal health actions from the women's perspectives can reduce their risk.

Concerns about fetal health and well-being, particularly genetic abnormalities, were very common among our participants, as they have been found to be in other studies [16,32,33,40]. Although having several fetal surveillance tests helped reassure women about their baby's health, as Baillie et al. (2000) have noted, for a number of women, feelings of anxiety may remain throughout the pregnancy [41]. Hoping for a desirable pregnancy outcome despite having higher perceived risk and anxiety levels was evident in our data. This discrepancy may lead to feelings of uncertainty. Sun et al. (2008), in a qualitative study of women of AMA, reported similar ambivalent and conflicted feelings, characterized by apparent pleasure and hidden fear [42]. This attitude has been referred as a “jubilant apprehension” by Yuan et al. (2000) that describes feelings of great joy and satisfaction, but also worry about childbirth outcomes [40].

Feelings of anxiety were described vaguely by our participants and there were notable variations among participants in expressing and wording their anxiety. This attitude may make the detection of anxiety in these women a challenge. There is evidence that antenatal anxiety is very prevalent and can increase the odds of postnatal depression [43]. Therefore, identification of women with anxiety is crucial so that effective interventions can be targeted appropriately. Healthcare providers should be aware that some women with high levels of anxiety tend to use different terms to communicate these feelings. Although creating a relaxed environment and establishing a non judgmental communication pattern in prenatal care visits may be beneficial for anxious women in disclosing their actual feelings about potential risks, there is growing evidence suggesting that prenatal screening should also include screening for both depression and anxiety [44-46]. We support this perspective and believe that using a reliable screening tool to assess anxiety in pregnant women may be useful to identify women who would benefit from strategies to reduce anxiety. A recent study in Australia demonstrated that the anxiety subscale of the Edinburgh Postnatal Depression Scale might be a reliable measure to screen antenatal anxiety [45].

Women experienced a decrease in their perceived risk with advancing gestational weeks, which could be due to women becoming adapted to the state of being pregnant or becoming more positive about the outcome of their pregnancy as it advanced. An uncomplicated pregnancy and favorable screening results may contribute to decreased perception of risk over the course of pregnancy as well. A decline in worry about the baby’s health from early pregnancy to the postpartum period has been reported in previous research [47].

Consistent with research in other fields, our results demonstrated that pregnant women's personal or vicarious experience with a risk may increase the psychological availability of the risk and consequently, its perceived probability [48,49]. In fact, being familiar with risk, through researching information or indirect experiences, may contribute to women’s risk perception. In the literature, the perceived characteristics of the risk such as prevalence, controllability, preventability, and seriousness along with the availability and representativeness heuristics are important elements in constructing risk perception [35,50]. Our findings point to the predictability of risk as a critical characteristic of risk in which predicting the risk and expecting it influenced participants' risk perception. A risk that is expected may be perceived as less risky than an unexpected risk, suggesting that clear communication is essential to help women have realistic expectations about their individual risk. A qualitative study by Patterson (1993) demonstrated that an unexpected shift in health situation or pregnancy outcomes was identified as a high risk condition by pregnant women, while the expected changes were considered as no risk [51]. One explanation is that anticipating the risk and being prepared to deal with it may increase a woman's perceived control and, consequently, decrease her risk perception.

While discrepancies between pregnant women’s and health care providers’ appraisals of risk have been documented in previous research [52-54], what appears to be less emphasized is the influence of health care providers' attitudes towards the risk on pregnant women's risk perception. Several women in this study reported not having any risk communication with their health care providers that focused on age as a risk factor. This has been interpreted by most participants as there not being any serious concern and may imply that pregnant women trust their health care provider’s opinion. In this regard, Heaman et al. (2004) reported that women with or without pregnancy complications rely on their health care providers in assessing risk status. A few participants reported negative risk communication with their health care providers. These participants also had higher anxiety and concern about the well-being of their fetus and pregnancy outcomes. Whether risk communication patterns can increase the anxiety or whether anxiety itself will alter women's interpretation of risk communication is not clear and needs further research. In literature, a link between maternal depression and higher perception of teratogenic risk has been reported [55].

### Implications

In the experience of our participants, any emphasis on the mother’s good health by their care providers was described as reassuring. Conversely, negative messages about age by purely emphasizing pregnancy risks associated with AMA were described as very destructive and challenging, particularly for women with high levels of anxiety. These findings indicate that although offering risk information is part of the risk communication process, the woman’s mental health should be considered to avoid unnecessary stress.

Comparison was a common risk appraisal strategy among our participants. However, women’s evaluations were distinct from those of health professionals in that the participants often compared their risk to a known population such as their family members, friends, or stories from real people, which is entirely different from risks determined by epidemiological studies using population data. This disjuncture stems at least in part from the fact that pregnant women’s understanding of risk is mostly based on their life experiences [31,56], suggesting that real stories can alter risk perception. This finding has important implications for practice and public health education. Risk must be tangible in order to be recognized and potentially addressed by women. Interventions to decrease unhealthy behaviors should target this concept to alter risk perception. As Williamson & Weyman (2005) suggested when individuals have less experience or knowledge of risks, the media (e.g., documentary movies) can play an important role in increasing their understanding of those risks [57].

One of the strengths of the study was the addition of quantitative measures (i.e., perception of pregnancy risk, anxiety, and perceived control) and the inclusion of a variety of perspectives from women of AMA with varying levels of risk perception, anxiety and perceived control. This diversity in the sample allowed for the documentation of variations in risk appraisal and also the identification of important issues that were common across participants. Another major strength was the contemporaneous exploration of risk perception during the pregnancy. It is important to consider the following limitations when interpreting and applying the results. First, our sample was representative of a middle class and married population; these characteristics may limit the generalization of the results. This is a small study based on 15 interviews in one geographical area in Canada; therefore, the results may not be generalizable to other populations. Finally, we recognize that selection bias may have been introduced as a purposeful sampling method was employed and those who volunteered to participate might have been different from those who did not.

## Conclusion

Pregnancy at age 35 years or older within a healthy context and in the absence of other risk factors was perceived as a low risk pregnancy by the majority of our participants. However, in the presence of risk factors such as pregnancy complications, limited physical activity, unfavorable screening tests results, previous poor reproductive history, and anxiety, the risk associated with age was highlighted, and women were inclined to recognize their age as a risk factor for their pregnancy. This study adds to the literature on perception of pregnancy risk by identifying several factors that influence the perception of pregnancy risk. Understanding of these influential factors may help health care providers who care for pregnant women aged 35 years or older to gain insight into their perspectives on pregnancy risk and improve the effectiveness of risk communication strategies with this group.

## Competing interests

The authors have no conflicts of interest to disclose.

## Authors’ contributions

HB and MH conceived and designed the study. KD and ST provided important feedback and suggestions in designing the study. HB acquired the data. All authors contributed to the analysis and interpretation of data. HB drafted the manuscript, and MH, KD, and ST revised it critically for important intellectual content. All authors read and approved the final manuscript.

## Pre-publication history

The pre-publication history for this paper can be accessed here:

http://www.biomedcentral.com/1471-2393/12/100/prepub
